# Neuronal Ensembles Organize Activity to Generate Contextual Memory

**DOI:** 10.3389/fnbeh.2022.805132

**Published:** 2022-03-15

**Authors:** William D. Marks, Jun Yokose, Takashi Kitamura, Sachie K. Ogawa

**Affiliations:** ^1^Department of Psychiatry, University of Texas Southwestern Medical Center, Dallas, TX, United States; ^2^Department of Neuroscience, University of Texas Southwestern Medical Center, Dallas, TX, United States

**Keywords:** hippocampus, contextual fear conditioning, entorhinal cortex, neural circuits, memory engram

## Abstract

Contextual learning is a critical component of episodic memory and important for living in any environment. Context can be described as the attributes of a location that are not the location itself. This includes a variety of non-spatial information that can be derived from sensory systems (sounds, smells, lighting, etc.) and internal state. In this review, we first address the behavioral underpinnings of contextual memory and the development of context memory theory, with a particular focus on the contextual fear conditioning paradigm as a means of assessing contextual learning and the underlying processes contributing to it. We then present the various neural centers that play roles in contextual learning. We continue with a discussion of the current knowledge of the neural circuitry and physiological processes that underlie contextual representations in the Entorhinal cortex-Hippocampal (EC-HPC) circuit, as the most well studied contributor to contextual memory, focusing on the role of ensemble activity as a representation of context with a description of remapping, and pattern separation and completion in the processing of contextual information. We then discuss other critical regions involved in contextual memory formation and retrieval. We finally consider the engram assembly as an indicator of stored contextual memories and discuss its potential contribution to contextual memory.

## Introduction

We have all experienced the recall of a specific memory when we are exposed to a similar situation; the feeling of a warm breeze on a beach might remind you of a summer day from your childhood when you enjoyed swimming with your family and a barbecue. It occurs because the target (the act of swimming with your family in this case), was memorized alongside multiple types of information that occurred at that moment (a warm breeze on the beach in this case). While we may focus on a specific event or person in the moment, many other pieces of information occurring simultaneously around the target become enmeshed in memory formation. Those other information streams can serve as a hint to facilitate recall of the target memory. Together, these are referred to as the “context” (Godden and Baddeley, 1975; Smith, 1979; Smith et al., 2004; Manns and Eichenbaum, 2009; McKenzie et al., 2014; Robin et al., 2018; Libby et al., 2019; de Voogd et al., 2020). Context can shape our decisions and our recall processes (Godden and Baddeley, 1975; Smith, 1979; Smith et al., 2004; Robin et al., 2018; de Voogd et al., 2020), and has been shown to be an important first step in processing and rebuilding of episodic memories, helping to streamline object representations (Manns and Eichenbaum, 2009; McKenzie et al., 2014; Libby et al., 2019), and has a role in the determination of motivation and valuation of actions and items (Zeithamova et al., 2018). Context includes external information and internal states (Spear, 1973; Bouton, 1993; Rudy et al., 2004; Maren et al., 2013). In laboratory observations of contextual learning, the external components collected by sensory systems such as visual information (e.g., a color of paint on the wall of a room, dark or brightness of light), odor, sound, and touch (e.g., texture of a floor) contribute to the formation of spatial-contextual information for animals. Thus, the external components prove easier to manipulate, and are often used as the primary means of modifying contexts. We can describe the context as the specifics of a place that are not the place itself. For example, it can be understood that the context changes if the color of paint on the walls in the room is changed, but the space of the room itself does not. The Internal elements can include emotions (e.g., happiness, fear, sadness, and anger etc.) and hormonal states such as hunger or stress experienced within the situation. These internal sorts of contextual stimuli are important; just as ones experience of a situation may vary with the context of an environment (dark vs. light) for example, ones experience of an event may differ depending on whether one is stressed or calm, or whether one is angry. The effects of these internal states can predispose a circuit to be more or less responsive to a given cue (Jezek et al., 2010; Maren et al., 2013; Tye, 2018). In animal behavioral models, there are various types of behavioral experiments in which an animal uses these “contextual” data to perform a task. Broadly, multiple tasks in which animals use place memory can be included as part of a context dependent memory paradigm such as contextual fear conditioning (CFC), passive avoidance, mazes, open field experiments etc. In this review, we focus primarily on CFC paradigms which are one of the most common behavioral tasks for assessing contextual memory, and highlight the brain regions that are involved in contextual memory processing. Among the many brain regions involved in contextual memory, the EC-hippocampal network has been widely studied across a number of behavioral paradigms, and is known to have a strong role in episodic memory formation. As a result, a great deal is known about the physiological processes that support learning and memory within this region (Scoville and Milner, 1957; Mahut et al., 1982; Kim and Fanselow, 1992; Phillips and LeDoux, 1992; Frankland et al., 1998; Eichenbaum, 2000; Tulving, 2002; Sutherland et al., 2008; Kitamura et al., 2012; Pilkiw et al., 2017). To that end, we begin with a main focus on the EC-HPC network as the most well studied region involved in contextual memory before shifting our focus to the large array of associated regions. We then discuss the contributions of engram assemblies both within and outside of the EC-HPC network to context-dependent memory.

## Contextual Memory

### Contextual Fear Conditioning

**FIGURE 1:**
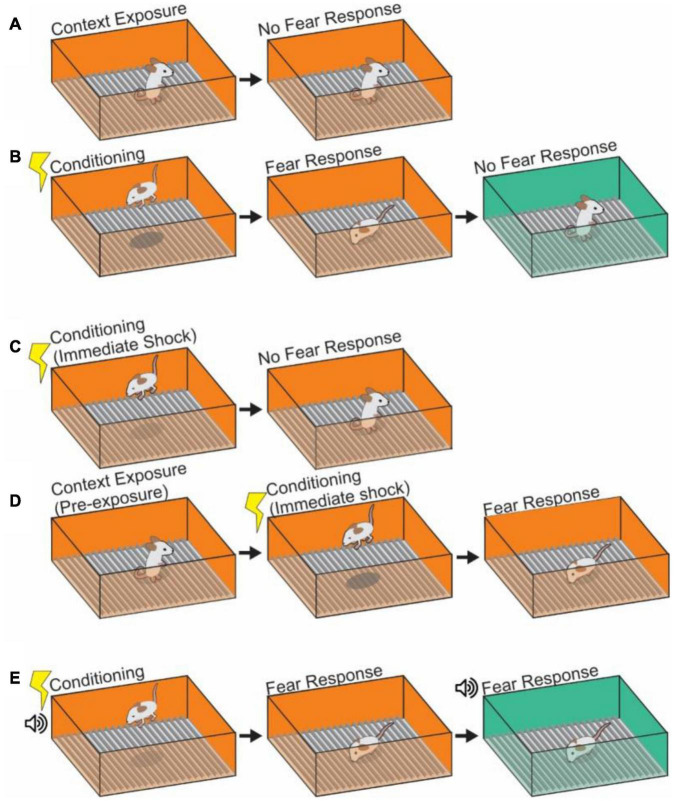
Depictions of contextual learning. **(A)** The exposure of an animal to a context results in learning of that context. However, in the absence of any frightening stimulus, no fear response will occur to the neutral context. **(B)** Pairing of a shock with a contextual stimulus will cause fear responses upon return to that conditioned context, indicating a pairing of shock memory with memory of the contextual cues. Subsequent placement in a second neutral context will not provoke a fear response. **(C)** Depiction of an immediate shock protocol. A mouse which is shocked immediately upon being placed in a context and removed after will not pair shock learning with context as insufficient encoding of the context itself has occurred. **(D)** Pre-exposure to a context will alleviate the failure to learn after immediate shock. Animals pre-exposed to the same or similar context 24 h prior to immediate shock protocol will associate the shock with the context. **(E)** Auditory fear conditioning can be added to create a more complex contextual pairing in which the fear response is tied both to the visual context and the auditory context (the tone). The tone can cause a fear response in a neutral context and generalization of fear to that context.

A common test used to assess contextual memory processes is the Pavlovian contextual fear conditioning (CFC) paradigm. An animal (a mouse or rat in most cases) is put into a conditioning chamber (conditioned stimuli, CS) which is designed to signal the delivery of an electrical foot-shock (unconditioned stimuli, US), and learns its specific context as paired with shock. The duration of freezing behavior is measured as an outcome when the animal is reintroduced into the same context vs. a novel context (Figures 1A,B). An animal shows freezing behavior in the conditioned context if an animal has formed a contextual fear memory following the association of a previous painful experience (Pavlov, 1927; Kim and Fanselow, 1992; Phillips and LeDoux, 1992; Frankland et al., 1998; Amaral and Lavenex, 2007; Sutherland et al., 2008; Maren et al., 2013; Kitamura et al., 2015, 2017). In order to learn the context, animals must form a representation of the context first.

### Immediate Shock and Context Pre-exposure

Previous studies demonstrated that the exposure time to the context chamber itself is crucial for context memory formation (Fanselow, 1986; Wiltgen et al., 2006). The experiments show that an animal does not learn the relationship of context and shock when a shock is presented immediately (less than 24 s) after animals are placed into a novel context (Figure 1C). This is referred to as the “immediate shock” effect. This phenomenon suggests that a certain amount of time processing the novel context, including texture, visual, auditory and sensory information in the environment, is necessary to form a contextual memory (Blanchard et al., 1976; Fanselow, 1986, 1990; Wiltgen et al., 2006), with longer durations spent in a context resulting in stronger unified representations of the context against which to form associations (Bae et al., 2015). Performing a pre-exposure to the context, in which an animal is habituated to the context in the absence of US (shocks) for 24 h before the US application works for the association between the context and shock even with immediate shock conditioning (Fanselow, 1990; Rudy and O’Reilly, 2001; Ohkawa et al., 2015). In this case an animal shows a significant level of freezing behavior in the context after the pre-exposure experience and following US conditioning (Figure 1D). This result indicates that pre-exposure to the shock chamber facilitates later association between the context and shock. Interestingly, several studies suggest that the degree of difference between the contexts may play a role in this context-shock association; when applying a pre-exposure and immediate shock across different, but closely related contexts it is possible to generate a false (generalized) fear memory (Rudy and O’Reilly, 1999; Bae et al., 2015; Lingawi et al., 2018; Libby et al., 2019), while an animal given pre-exposure in a vastly different context does not create false memories following US conditioning. The degree of similarity between two contexts (in this case the pre-exposure and immediate shock chambers) and the amount of time spent in a context both appear to be critical in determining how generalized or specific a fear memory can be. These interactions appear to be very sensitive due to the underlying complexities of the supportive processes (Lingawi et al., 2018; Sevenster et al., 2018), highlighting the need for increased study of the underlying mechanisms.

### Cue Associated Contextual Learning

Beyond static contextual cues, discrete and salient sensory presentations of CS also become associated with the presentations of US through “cue associated” learning (Phillips and LeDoux, 1992; Goosens and Maren, 2001; Kim et al., 2013; Pellman and Kim, 2016). In auditory-cued fear learning (Figure 1E), for example, animals can learn through CS-US association that a neutral tone presentation (as CS) in a particular context (chamber A) is contingently paired with foot-shock (as US) that induces freezing behavior. After the conditioning, the animal shows freezing behavior when the tone is applied even in a different chamber (chamber B) (Curzon et al., 2009; Maren et al., 2013). A discrete CS, like this tone representation, that is separated from the context allows for the separation of which processes are related to fear memory itself, and which are due to the contextual element, as well as defining the linkage between the two processes (Anagnostaras et al., 2001; Rudy et al., 2004). The brain regions that contribute to cued fear conditioning can vary depending on the intensity of CS and US presentation, timing, and duration. Contextual fear memory (and fear memory in general) is known to be largely mediated by the amygdala (Phillips and LeDoux, 1992; Goosens and Maren, 2001; Kim et al., 2013; Pellman and Kim, 2016), and having a means to assess the contribution of amygdala to CFC by associating a tone has generated greater insight into the processes by which contextual and other cued information streams are combined in the HPC, for example, that fear association with a tone is sensitive to lesion in of the insular cortex, while fear association with a context is not, as well as the identification of the involvement of other brain regions in CFC including auditory cortex, Retrosplenial cortex (RSC) and thalamus (see detail in section “Other Brain Areas Involved in Contextual Memory”) (Phillips and LeDoux, 1992; Fanselow and LeDoux, 1999; Brunzell and Kim, 2001; Kochli et al., 2015; Kwapis et al., 2015; Bergstrom, 2016; Pollack et al., 2018; Chaaya et al., 2019). Depending on how the cued CS is presented, auditory-cued fear conditioning can be further subcategorized as delay fear conditioning or trace fear conditioning: Delay fear conditioning refers to a delay procedure in which the CS (Tone) is followed by US (foot-shock) and those presentations are temporally contiguous (Phillips and LeDoux, 1992; Fanselow and LeDoux, 1999). It is thought that the lateral amygdala receives tone information from the auditory cortex and auditory thalamus. Trace fear conditioning, by contrast, has a time interval introduced between the termination of the CS and the onset of the US (Clark and Squire, 1998; Buchel and Dolan, 2000; Misane et al., 2005; Kitamura et al., 2014; Kochli et al., 2015; Yokose et al., 2021). This paradigm requires an animal to track the structure of the temporal gap between CS and US presentations. This is controlled by specialized projections from MEC to HPC (McEchron et al., 1999; Quinn et al., 2002; Chowdhury et al., 2005; Gilmartin and McEchron, 2005; Suh et al., 2011; Kochli et al., 2015) (see section “Neural Circuits for the Contextual Memory in the Entorhinal Cortex-Hippocampal Formation”). As we described above, context information is composed of multiple elements in an animal’s environment. Here, we stop to ask if there is any systematic rule that allows for the individual contextual information components to be recorded in contextual memory. Researchers have been interested in this question for a long time, and have proposed mechanisms to explain the phenomenon.

### The Hierarchical Nature of Contextual Information

Richard Hirsh hypothesized that contextual cues have a hierarchical property rather than a universal equivalence (Hirsh, 1974; Nadel and Willner, 1980). Hirsh’s hypothesis stated that context was i) hierarchical, operating in the background relative to other memories, and ii) acted to serve as an index system for other memories (Hirsh, 1974; Nadel and Willner, 1980), which contrasted with earlier ideas that treated context as an independent CS which was functionally equivalent to any other CS (Rescorla and Wagner, 1972; Wagner and Rescorla, 1972; Odling-Smee, 1975). In support of Hirsch’s theory, even in context-independent tasks, contingent context learning occurs; altering the presented context after the initial trial attenuates conditioned responses to the CS as well as latent inhibition (For example, animals conditioned to respond to air puffs delivered to the eyes (CS) in context A showed reduced responses to the same CS in context B), suggesting that animals are passively learning and integrating the contextual cues even though they are not directly relevant to the learned task (Penick and Solomon, 1991; Honey and Good, 1993), and indicating that context does not behave like a normal CS as previously thought. In a number of associative learning studies, contextual stimuli can be seen behaving as “occasion setters,” which, instead of becoming directly associated, modulate other associative linkages (Bouton and King, 1983; Bouton and Peck, 1989; Bouton and Brooks, 1993). For example; if establishing contextual fear conditioning in a given context (context A) paired with a tone, and performing tone-extinction relearning in a second (context B) with a tone, animals shows less freezing when re-exposed to context A (Bouton and King, 1983; Bouton and Peck, 1989; Bouton and Brooks, 1993), showing that the animal’s behavioral selection is dependent on the contextual presentation, that is, the suppression of responding that results from presentation of the extinction context works as a negative occasion setter whose role is to disambiguate the current meaning of the conditioned context. Notably, this disruption is affected by the context presentation itself, not the associative strength or conditioning efficiency (Bouton and King, 1983; Bouton and Peck, 1989; Hall and Honey, 1989; Kaye and Mackintosh, 1990). In this way, the associative strength of the CS is subject to selection by the occasion setting function of contextual stimuli; a secondary contextual association inhibits the original association once a novel association is formed in a secondary context, acting as a forced selector depending on the contextual presentation (McCloskey and Cohen, 1989; Swartzentruber, 1991), functionally setting the occasion, or in other words, selecting a behavioral state to operate in relative to and dependent on particular presentations of external stimuli. In light of these findings, it can be inferred that, due to the sensitivity of CS-US associations to contextual presentations, contextual stimuli must be accounted for before the association happens, supporting the idea that contextual activity is hierarchical and can be combined with other data streams (Harris et al., 2000; Chang and Liang, 2017). Thus, it appears that context is a collection of multiple background data that can operate as a map and modify other maps (Hirsh, 1974; Nadel and Willner, 1980).

## The Role of the Entorhinal Cortex-Hippocampal Formation for Contextual Memory

**FIGURE 2:**
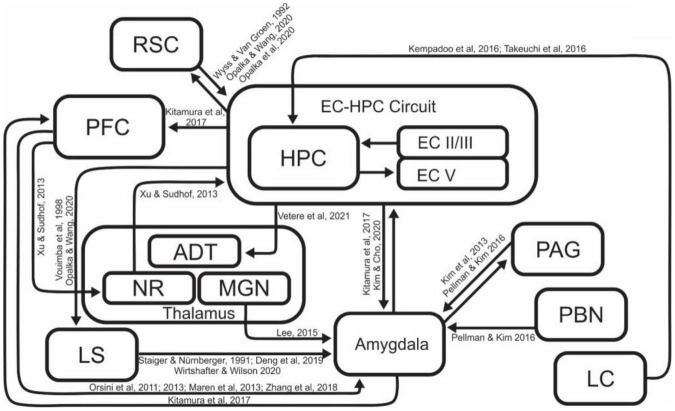
A schematic diagram of the brain regions and related connections involved in contextual fear learning. While several regions feature more prominently than others in this process, a number of regions are involved in generating and refining contextual representations and associations. ADT, Anterodorsal Thalamus; EC, Entorhinal Cortex; HPC, Hippocampus; LC, Locus Coeruleus; LS, Lateral Septum; MGN, Medial Geniculate Nucleus; NR, Nucleus Reuniens; PAG, Periaqueductal gray; PBN, Parabrachial Nucleus; PFC, Prefrontal Cortex; RSC, Retrosplenial Cortex.

Many different brain regions are involved in contextual fear memory acquisition, consolidation, and retrieval (Figure 2). In particular, there have been many reports on the necessity of EC-HPC formation in contextual memory processes. The HPC has been shown to be involved in the differentiation of contexts (Frankland et al., 1998), and integration of contextual information with learned behaviors and responses (Good and Honey, 1991; Honey and Good, 1993; Freeman et al., 1997; Smith et al., 2004; Kim et al., 2012). Lesion of the HPC disrupts CFC (McEchron et al., 1999) in a manner that is time sensitive, with recently acquired memories being destroyed by lesion, and longer term contextual memories being undamaged (Kim and Fanselow, 1992; Anagnostaras et al., 1999; Lee et al., 2017). Pharmacological inactivation of the dorsal HPC impairs context-dependent memory recall (Corcoran and Maren, 2001; Barrientos et al., 2002). On the other hand, there are also reports that contextual fear memories can be acquired even in the absence of the HPC. These findings raise the question of how critical the HPC is in the learning and recall of contextual fear memory. Several groups have performed contextual fear conditioning in HPC lesioned rodents to demonstrate that animals could indeed acquire contextual memory even in the absence of the HPC, however, this hippocampal independent conditioning required more shock events or more learning sessions to account for decreased efficiency of acquisition (Wiltgen et al., 2006; Lehmann et al., 2009). Depending on conditions of the experiment, these hippocampal independent memories can be less robust; disappearing more quickly that those generated with the hippocampus intact (Zelikowsky et al., 2012), while other studies demonstrate that memories generated without the hippocampus can be robust, lasting as long as thirty days (Lehmann et al., 2009; Gidyk et al., 2021). These results suggest that the acquisition of contextual fear may have redundant or compensatory systems, potentially explaining why hippocampus independent learning requires more shock events or more learning sessions (Wiltgen et al., 2006; Lehmann et al., 2009; Chaaya et al., 2018). The brain has a robust ability to compensate for damage, and these reports are presumed to be the result of the ability to acquire contextual fear memory by supplementing the absence of the HPC with other brain regions to counterbalance the disruption of normal operations. Entorhinal Cortex (EC), a major contributor to the functionality of the hippocampal circuit, is also known to play a key role in contextual memory processes, and it is reported that the lesion of EC results in decreased contextual learning, but not avoidance learning (Freeman et al., 1997). Chemical inactivation of the MEC also leads to alterations in contextual reconsolidation and extinction, supporting the regions’ role in processing contextual information (Baldi and Bucherelli, 2014). These two regions (EC-HPC) are part of a tightly interconnected network, which is collectively the most studied region involved in processing of contextual and episodic memories.

Taken together, these reports highlight that the EC-HPC formation plays a core role in contextual memory processes with a broader network supporting the expression of contextual memories following their initial formation. In the following sections, we mainly focus on EC-HPC formation as one of best studied contributing brain regions in the processes of contextual memory, and discuss their neuronal circuit connectivity and physiological mechanisms in relation to contextual memory. We describe other relevant brain regions, and how the individual components are involved in CFC as well.

### Neural Circuits for Contextual Memory in the Entorhinal Cortex-Hippocampal Formation

**FIGURE 3:**
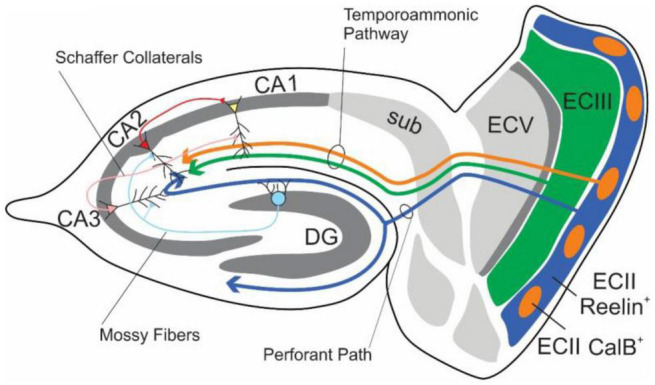
Schematic diagram of the major connections of the EC-HPC network. Axons from the EC layer III and CalB^+^ pyramidal cell clusters in EC layer II project along the temporoammonic pathway (ECIII/ECII CalB^+^ → CA1), which largely governs temporal features, The indirect pathway, which is involved in spatial and contextual learning, originates from Reelin^+^ stellate cells within EC layer II and projects to the classical tripartite synapse (ECII Reelin^+^ → DG → CA3 → CA1). Additional connections from DG to CA2, and CA2 to CA1 exist alongside the more heavily studied hippocampal pathways.

The EC-HPC formation have been considered to be crucial for learning and memory (Eichenbaum and Cohen(eds), 2004; Kitamura, 2017; Figure 3). In the main excitatory hippocampal network, there are parallel pathways referred to as the trisynaptic pathway (EC layer II→adentate gyrus (DG) →CA3 →CA1) and the monosynaptic pathway (EC layer III→CA1). The pathway From ECII to CA3 via the DG is understood to be critical in the creation of differential representations of contexts and spaces with high degrees of overlap, while the CA3-CA3/CA1 pathways are understood to be critical in the identification of contexts from partial cues (see section “Pattern Separation and Pattern Completion”).

Most physiological studies of contextual processing were carried out in the HPC, however, it was more recently that studies of the role of the EC, upstream of HPC, in contextual memory were undertaken. A great deal of the contributing mechanism was unknown. For example, it was unknown whether contextual information was already processed in a subset of EC cells or not, and what the key driver for routing of contextual information to HPC was. Originally, grid cells, which are critical for various aspects of spatial navigation, were “thought to provide a context-independent metric representation of the local environment” (Giocomo et al., 2011). Recently, the question of upstream drivers of contextual learning has been gaining more attention.

To elucidate circuits upstream from HPC, previous studies demonstrated with electrolytic lesion or pharmacological inhibition that the EC is necessary for contextual fear conditioning (Maren et al., 1997; Lewis and Gould, 2007). However, due to the lack of cell-type specific manipulation, it remained unclear how the EC contributes to the contextual fear conditioning. One important study began to examine the role of specific cell types within individual layers of medial entorhinal cortex (MEC) in contextual memory processing. In the section above, we describe trace fear conditioning, which contains a temporal gap between the end of the CS and the onset of US called the “trace period” or “trace interval.” It was revealed that the synaptic plasticity of CA1 pyramidal neurons is necessary for the representation of the trace period (Tsien et al., 1996; Huerta et al., 2000; Fukaya et al., 2003). A monosynaptic pathway from MECIII to CA1 pyramidal neurons was shown to be crucial for trace fear conditioning, demonstrated using MECIII mutant mice that had tetanus toxin light chain expressed in dorsal MECIII (Suh et al., 2011), and optogenetic inhibition of MECIII input to CA1 neurons (Kitamura et al., 2014). Further study by Kitamura et al. (2014) revealed that there are two distinct subtypes of projection neurons found in MECII, CalbindinD-28K (CalB)^+^ pyramidal cells and Reelin^+^ stellate cells, with the former projecting to interneurons in stratum lacunosum (SL) of CA1 area and the latter monosynaptically projecting to DG neurons (Kitamura et al., 2014). The inputs from the CalB^+^ pyramidal cell clusters in MECII suppress the excitatory MECIII input into the CA1 pyramidal neurons via feedforward inhibition achieved by activation of SL interneurons to control the strength and duration of trace fear memory (Kitamura et al., 2014; Yokose et al., 2021).

On the other hand, optogenetic inhibition of Reelin^+^ cells in MECII, which project to DG neurons, during memory acquisition or retrieval disrupted both formation and recall of CFC memory (Kitamura et al., 2015). Kitamura et al. (2015) applied cell type-specific *in vivo* Ca^2+^ imaging in both Reelin^+^ stellate cells and CalB^+^ pyramidal cells individually in MEC layer II, and found that Reelin^+^ stellate cells, but not CalB^+^ pyramidal cells, have context specific neural activity and drive context-specific CA3 activation and CFC memory (Kitamura et al., 2015). The context-specific responses in Reelin^+^ stellate cells were still observed when dorsal CA1 activity was inhibited by muscimol, a gamma-aminobutyric acid subtype A (GABA_A_) receptor agonist (Kitamura et al., 2015). This finding suggests that ensembles of Reelin^+^ stellate cells in MEC layer II are essential, carry full contextual information, and transfer it to HPC. These results demonstrated that Reelin^+^ cells are essential for the formation and recall of CFC memory, while CalB^+^ cells were found to be crucial for temporal association learning, demonstrating that the two excitatory MEC layer II inputs to the HPC have different, but complementary roles in episodic memory (Kitamura et al., 2014, 2015; Yokose et al., 2021). Importantly, optogenetic inhibition of the MECIII inputs into hippocampal CA1 has no effect on CFC memory, indicating that the hippocampal trisynaptic pathway, but not the monosynaptic pathway, is crucial for the CFC memory (Suh et al., 2011; Kitamura et al., 2014). Since the optogenetic inhibition of DG granule cells does not impair the contextual fear response itself, but does increase the level of freezing in an unconditioned context (Kitamura et al., 2015), the direct MECII to CA3 pathway would be a main driver for contextual fear memory, while MECII-DG-CA3 pathway would be more specialized for contextual discrimination/pattern separation. These optogenetic experiments were temporally targeted during the conditioning phase. A recent work, however, has demonstrated the necessity of MEC inputs into HPC immediately after conditioning has concluded (Kang and Han, 2021). It was also revealed that the subpopulation in MEC which are not grid cells drive the context specific activity. Diehl et al. (2017) showed that approximately 95% of cells in the superficial layers of MEC were identified as spatially active cells spanning multiple functional categories including populations of grid cells, non-grid cells, and border cells. The non-grid spatial cells responded to contextual box manipulations with reorganized spatial firing, while the grid cell activity of the EC did not exhibit contextual sensitivity. Although context specific-activity and necessary circuits for contextual memory have been found in the projections from MEC II Reelin^+^ stellate cells, it still remains unclear exactly how the HPC processes and integrates contextual information.

These neurophysiological observations, discussed further in the sections “Remapping” and “Pattern Separation and Pattern Completion” below, open up further questions of differential and local processing modalities between these regions that can independently shape spatial and contextual representations as they form. Within the HPC, local networks also contribute to the control of CA subfield pyramidal neuron activity (Pelkey et al., 2017). HPC GABAergic interneurons have been shown to have a role in CFC expression (Gilmartin et al., 2012; Almada et al., 2013). Overactivation of GABA receptors has been shown to impair CFC without impairment of the ability to recall a shock in a separate, context independent paradigm (Chang and Liang, 2012; Misane et al., 2013). Additionally, specific GABA receptor subunits on pyramidal cells have been tied to different aspects and overall intensity of contextual learning (Lehner et al., 2010; Moore et al., 2010; Engin et al., 2016, 2020). These findings suggest that the local networks play a highly complex and input specific role in the formation of contextual memories. Moreover, outside of the EC-HPC network, there are multiple reports about the contributions of other brain regions involved in contextual memory formation which may directly modulate EC-HPC network activity (see section “Other Brain Areas Involved in Contextual Memory” below). Further study will be expected to understand neural circuit mechanisms for the formation and recall of CFC memory.

### Physiological Representations in Contextual Memory

#### Remapping

The EC-HPC network is most known for its function in the processing and encoding of spatial information (O’Keefe and Nadel, 1978; Eichenbaum et al., 1999), with individual pyramidal cells able to act within an ensemble specific to a particular location and function as “place cells” which are generated in “place fields” (O’Keefe and Dostrovsky, 1971; O’Keefe et al., 1998). The functional activity of the place cells essentially allows for the mapping and representation of multiple discrete spaces by the HPC (O’Keefe and Conway, 1978; Kubie and Ranck, 1983; Skaggs and McNaughton, 1996; Louie and Wilson, 2001; Dragoi and Tonegawa, 2011; Kubie et al., 2020). Neuronal firing activity patterns within an ensemble change along with the changes in environment and reorganized cell ensembles represents the position of animal. This ensemble activity change is called “remapping” and is categorized differentially based on its associated representation.

The first report of remapping was observed by O’Keefe and Conway (1978), who analyzed the firing activity of place cells in rats on an elevated platform and on a T-maze. Across the two different environments, a population of place cells fired in the first environment but not second, while another set of cells fired in the second but not the first. This phenomenon was termed “global mapping” (O’Keefe and Conway, 1978), reflecting the discrete separations between environments as two discrete place cell ensembles.

By contrast, when spatial cues or other contextual modifiers are only partially altered, a subgroup of place cells change their firing patterns, creating a new ensemble consisting of some of the original place cells and some new place cells, termed “partial remapping” (Muller et al., 1987; Bostock et al., 1991; Kubie and Muller, 1991; Markus et al., 1995; Kubie et al., 2020). Anderson and Jeffery (2003) demonstrated this in the rat CA1 using compound contexts comprised of two differently colored boxes paired with two different odors. The majority of neurons changed their firing fields across varying combinations of both box and odor, but some neurons responded to the color or odor changes alone. This demonstrated that altering certain features of the environment can cause a partial shift in the population of active place cells.

Additionally, “rate remapping” can occur in which the firing field of individual neurons remains the same, but their firing frequency changes (Leutgeb et al., 2005; Kubie et al., 2020). Leutgeb et al. (2005) demonstrated this in ensemble activity recordings from dorsal CA1 and CA3 in freely moving rats. They examined the neuronal firing pattern and rate in two experimental conditions; either a variable cue in a constant place; or identical cues in varied places. Rate remapping was most evident in CA3, with particularly large firing rate changes and little change in firing field, particularly in the latter case. This is of particular interest, as CA3 is thought to be where contextual representations are built prior to their storage in CA1 (Mizumori et al., 1999; Wintzer et al., 2014; Zhao et al., 2020).

The different remapping modalities can be reflective of differential neuronal processing modalities. Global remapping in the HPC occurs almost immediately, consistent with the magnitude of the change between environments (Kentros et al., 1998; Hayman et al., 2003; Wills et al., 2005), and differential spatial ensembles stabilize quickly after animals are introduced to the novel environment (Wilson and McNaughton, 1993; Frank et al., 2004). However, ensembles stabilize more slowly in CA3 than in CA1 after exposure to a novel space (Leutgeb et al., 2004). This suggests that hippocampal representations are formed independently and utilizing unique local processing methods. The differential processes between CA1 and CA3 may be modulated by CA2 activity which has a critical role in reconciling differences between incoming contextual data (CA3) and stored contextual data (CA1) by facilitating remapping of the representations in CA3 and CA1 (Wintzer et al., 2014; Boehringer et al., 2017). The flexibility gained by remapping combined with the ability of the HPC to rapidly generate new ensembles (Ziv et al., 2013) allows for quick encoding of a context with ensemble activity (Dragoi and Tonegawa, 2011, 2013), and a high level of complexity in the encoding and integration of contextual information with other data streams (Chang and Liang, 2017; Gulli et al., 2020).

#### Pattern Separation and Pattern Completion

The prediction of place fields or generation of associational assemblies in CA1 are not directly correlated to CA3 or DG activity, meaning that somewhere along the information path through the hippocampus, divergent mechanisms for generating ensembles from contextual data, and reactivating previously generated ensembles from observed stimuli must exist; these are referred to as pattern separation and pattern completion, respectively (Leutgeb et al., 2007; Colgin et al., 2008; Sahay et al., 2011a; Rolls, 2013; Lee et al., 2020; Carrillo-Reid, 2021).

Pattern separation is the process of differentiating similar input patterns into distinct outputs to prevent incorrect associations. This process is believed to occur at the connection between DG and CA3. The idea that pattern separation takes place along this pathway is associated with the large number of DG granule cells and their relatively sparse activity, and the fact that DG-CA3 synaptic connections tend to have a low redundancy (Marr, 1971; O’Reilly and McClelland, 1994; Treves and Rolls, 1994; Leutgeb et al., 2004, 2007; McHugh et al., 2007; Bakker et al., 2008; Wintzer et al., 2014). The DG has a unique form of neural circuit plasticity, in which adult-born DG cells make novel and sparse connections to CA3. Studies of animals with either impaired adult neurogenesis (Altman and Das, 1965; Schlessinger et al., 1975; Seki and Arai, 1993; Eriksson et al., 1998) or blockade of DG-CA3 synapses demonstrated that contextual discrimination is not dependent on extant/stable mossy fiber inputs to CA3 (Nakashiba et al., 2012). Rather, this function is driven by newly generated granule cells and their novel connection with the network (Clelland et al., 2009; Kitamura et al., 2009; Scobie et al., 2009; Creer et al., 2010; Arruda-Carvalho et al., 2011; Sahay et al., 2011b; Nakashiba et al., 2012; Gage and Temple, 2013; Kitamura and Inokuchi, 2014; Alam et al., 2018; Terranova et al., 2019).

In animals with synaptic transmission-deficient CA3 pyramidal cells, contextual fear conditioning is impaired (Nakashiba et al., 2008), indicating that the CA3-CA1 and CA3-CA3 circuits have a central role in the processing of contextual information. In particular, the recurrent CA3-CA3 network is known to be crucial for pattern completion which is the process of reconstructing completed representations from parts of stored components (Guzman et al., 2016). Recurrent CA3-CA3 synapses appear to have a sparse interconnectivity, suggesting high specificity in their connections rather than a broad activation. This could account for much of the processing needed to complete a pattern from a partial cue; a small subset of recurrent connections activated by an even smaller subset within (Leutgeb et al., 2007; Rolls, 2013; Guzman et al., 2016). These connections are critical in fast object-place recall, as one may expect in a situation where an animal is exposed to a particular set of external stimuli and needs to determine which behavioral state is appropriate. As such, this necessitates rapid reconstruction from partial cues as a matter of efficiency (Nakazawa et al., 2002, 2003; Gold and Kesner, 2005; Leutgeb and Leutgeb, 2007). It is possible that this process, if not tightly controlled enough or presented with ambiguous cues might be responsible for the generation of generalized contextual fear memories discussed earlier (Rudy and O’Reilly, 1999; Bae et al., 2015; Lingawi et al., 2018). The pattern completion process can be understood as the mapping and remapping activity of the CA3/CA1 subfields; a novel field being formed, or a field being recalled in response to a (partial or complete) set of discrete contextual cues (Nakazawa et al., 2002, 2003; Gold and Kesner, 2005; Leutgeb and Leutgeb, 2007).

## Other Brain Areas Involved in Contextual Memory

In addition to the EC-HPC network, a number of other brain regions were reported to contribute to contextual memory formation (Figure 2), either by contributing to the acquisition and retrieval of context memory directly, or regulating EC-HPC networks.

The basolateral amygdala (BLA) has been demonstrated to play a role in contextual fear conditioning, primarily by generating the emotional valence associated with the context-shock pairing. HPC interacts with the BLA via MECVa to encode contextual information during encoding of contextual fear memory. Not only the dorsal HPC (dHPC), but also the ventral CA1 (vHPC) projections to the Basal amygdala, paired with aversive stimuli, contribute to encoding conditioned fear memory (Kim and Cho, 2020). Hippocampal projections to the amygdala are involved in recent memory recall and association of contextual stimuli with pain. BLA receives foot-shock US information via the ascending pain pathways including the periaqueductal gray (PAG) and the parabrachial nucleus (PBN) and auditory CS information via thalamus (Kim et al., 2013; Lee, 2015; Pellman and Kim, 2016) and projects not only back to the HPC, but to the PFC (Kitamura et al., 2017).

Kitamura et al. (2017) demonstrated that in addition to receiving amygdalar input, PFC also receives contextual fear information from HPC via MECVa cells and that PFC engram cells are generated during the CFC encoding period. With optogenetic manipulation of both pathways, HPC-MECVa to PFC and BLA to PFC, it was revealed that both pathways are crucial for the generation and maturation of PFC engram cells following CFC, and are key for formation and recall of remote memory. Moreover, contextual activity in the PFC has been demonstrated to directly influence amygdalar activity (Orsini et al., 2011, 2013; Maren et al., 2013; Zhang et al., 2018).

Beyond the role of the Medial Geniculate Nucleus (MGN) in routing tonal information to the amygdala (Lee, 2015), the thalamic nuclei are also known to be involved in processing contextual information. Fear memories stored in PFC are either heightened or dampened in the nucleus reuniens (NR) of the thalamus prior to routing to HPC, resulting in a control of generalization of fear memory to differential contexts (Richter-Levin and Akirav, 2000; Xu and Sudhof, 2013; Rozeske et al., 2015). Additionally, a sparse inhibitory projection from CA3 to the anterodorsal thalamic nucleus is necessary for contextual fear memory retrieval at remote, but not recent time points (Vetere et al., 2021).

The Retrosplenial cortex (RSC) contributes to spatial navigation, head direction, as well as learning and memory for discrete cues such as auditory or visual stimuli. Lesion studies demonstrate that RSC processing contributes to configuration of contextual representation building (combining of elements). This is particularly interesting considering the previously demonstrated roles of RSC in hippocampal place field remapping (Wyss and Van Groen, 1992; Cooper and Mizumori, 2001; Mao et al., 2017; Todd et al., 2017; Alexander et al., 2020). Optogenetic inhibition of dHPC terminals in the RSC during memory acquisition causes impairment of subsequent memory performance (Opalka and Wang, 2020).

The Lateral septum (LS) is downstream from the HPC and has been implicated in various functions such as mood, motivation, anxiety, and spatial behavior, as reviewed in Wirtshafter and Wilson (2021). HPC terminal inhibition in LS during memory acquisition or retrieval tells us the dHPC-LS pathway plays a critical role in both contextual memory acquisition and retrieval (Opalka and Wang, 2020; Opalka et al., 2020). It thought that LS is important for matching context with behavior by taking contextually relevant information from the hippocampus and routing that information to the amygdala (Penick and Solomon, 1991; Staiger and Nurnberger, 1991; Deng et al., 2019; Wirtshafter and Wilson, 2021). Additionally, LS and full basal forebrain lesions can result in inappropriate pairing of context and movement response (Vouimba et al., 1998; Knox and Keller, 2016).

Recently the involvement of neuromodulatory inputs in contextual memory has begun to attract attention. It was reported that the Locus coeruleus (LC) tyrosine-hydroxylase-expressing (TH^+^) neurons project strongly to the HPC, and release dopamine. Optogenetic activation of LC TH^+^ neurons increased dopamine release into dHPC, promoting novelty associated spatial learning and memory (Kempadoo et al., 2016; Takeuchi et al., 2016). We expect further elucidation of relevant brain regions and understanding of the mechanism of contextual memory from the perspective of the entire brain.

## Engrams and Contextual Memory

Neural representations of context, dependent on the repeated activation of neural ensembles, remain stable over a period of time to facilitate recall and integration. It is understood that repetitive exposures to the same context activates the same set of cells as assessed using the expression of immediate early genes (IEGs) such as c-Fos, Arc (activity-regulated cytoskeleton-associated protein) or Zif268 (Wilson and McNaughton, 1993; Radulovic et al., 1998; Guzowski et al., 1999; Guzowski, 2002). German evolutionary zoologist Richard Semon first posited the term “engram” to describe a hypothetical physical substrate for memory storage (Semon, 1921; Schacter et al., 1978). While Semon’s idea was impossible to prove in the early twentieth century, recent technologies have made it possible to revisit his idea and the definition was updated to fit our modern understanding of memory. The following three criteria must be met for an event to be considered as engram (Josselyn, 2010; Tonegawa et al., 2015, 2018; Denny et al., 2017; Josselyn and Tonegawa, 2020). An engram (i) is an enduring and significant physical or chemical alteration to a neural network (ii) due to activity in a subset of neurons caused by episodic stimuli (iii) that can be reactivated following presentation of all or part of the original stimulus set, leading to memory recall. Technological advances have allowed for the targeting of individual cell subtypes by gene expression, which can include constitutively active proteins or more transient expression caused by neuronal activity (Guzowski et al., 1999; Guzowski, 2002; Han et al., 2007, 2009; Reijmers et al., 2007; Guenthner et al., 2013; Roy et al., 2017a).

**FIGURE 4:**
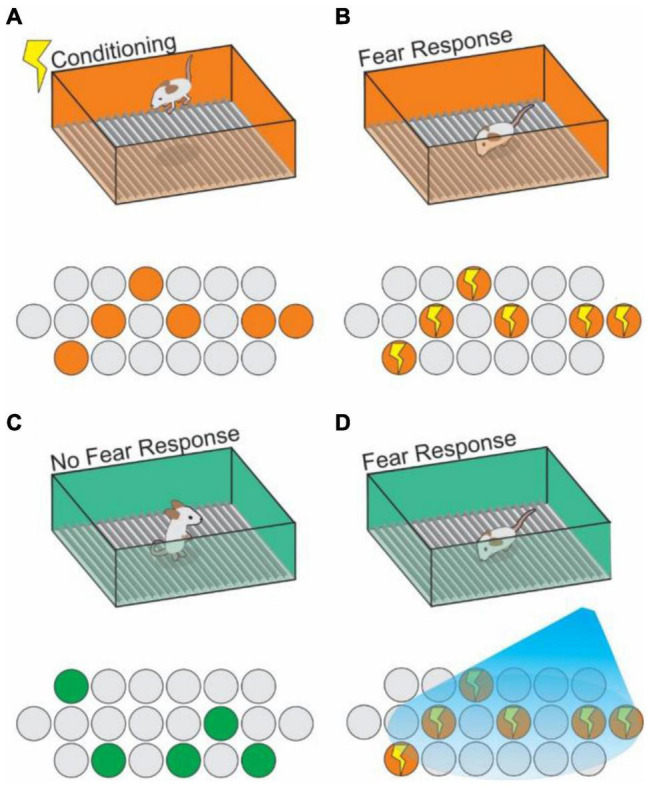
Context engram reactivation in the hippocampus can trigger fear memories. **(A)** An animal receives a shock in Context A (orange box) and generates a context specific ensemble (orange circles) into which the shock memory is tied (contextual fear conditioning). **(B)** Upon re-exposure to context A, the previously generated neural ensemble is activated (indicated by lightning bolts), and a fear response to the context is triggered. Using active cell tagging, the previous contextual ensemble is marked with an opsin for later reactivation. **(C)** The animal explores a novel context (Context B; green box), with no association to the shock training, learns the context and generates a new contextual ensemble (green circles) and exhibits no fear response. **(D)** Optogenetic reactivation of the Context A ensemble causes reactivation of the fear memory associated with Context A in the setting of Context B.

By utilizing IEG promoters to allow activity-dependent labeling of cells, and pairing this technique with optogenetically driven expression of opsins in previously activated cells, Liu et al., in 2012 made first observations of an “engram cell” under the modern definition (Reijmers et al., 2007; Liu et al., 2012). DG cells which had been active during CFC were targeted with ChR2, and stimulated with blue light at 20Hz were reactivated, and this reactivation artificially induced contextual memory recall without re-exposure to the original context information (Liu et al., 2012; Figure 4). Engram cells have since been found to have synapses with other engram cells resulting from the same memory event in the HPC (Choi et al., 2018). In this way, engrams can be further described as networks of individual engram cells which act together to store the components of memories, and reactivation of that network is both necessary and sufficient for recalling that encoded memory (Ryan et al., 2015; Tonegawa et al., 2015; Holtmaat and Caroni, 2016; Denny et al., 2017; Figure 4). Thus, we can understand an engram as the physical record of a representation, with the activated ensemble becoming reactivatable within a given set of stimuli (context) resulting in recall (Kelemen and Fenton, 2010; Jezek et al., 2011; Tayler et al., 2013). Importantly, engrams are distinctly encoded independently of other memory modalities (e.g., A spatial engram or a contextual engram) (Tanaka et al., 2018; Ghandour et al., 2019; Marks et al., 2021). Moreover, engram activation can even be sufficient to induce a false memory in the form of generalization of a fear response in one context onto a neutral context (Ramirez et al., 2013; Ohkawa et al., 2015; Oishi et al., 2019), or even to construct a representation of a context that has never been physically experienced (Garner et al., 2012). Kitamura et al. (2015) demonstrated how hippocampal engram cells are activated during memory recall. They found that the neural input from Reelin^+^ cells, but not CalB^+^ cells, in MEC drives contextual information into the HPC, is necessary for the formation and recall of CFC memory. They further showed that the neural input from Reelin^+^ cells in MEC is necessary for the reactivation of memory engram cells encoding CFC memory in the hippocampal CA3. These results indicate that the hippocampal trisynaptic pathway driven by Reelin^+^ cells in MEC layer II is crucial for CFC memory.

Recently, the question of when engram cells are formed during the process of memory acquisition and long-term storage has come into focus. Kitamura et al. (2017) found that engrams are actually formed simultaneously in the early stage of context memory acquisition in both the HPC and the medial prefrontal cortex (mPFC) where long-term memory is stored (Kitamura et al., 2017). This result went against the Standard Consolidation Model, which states that newly acquired episodic memories are initially stored in only HPC and then transferred to cortex as long-term memories, accompanied by “deletion” of HPC engrams with time. The mPFC engram cells which were generated in the early stage are not reactivated during the recent memory recall state but exist as “silent engrams” like those previously found by Ryan et al. (2015). These silent engrams become functionally mature with time, simultaneous with transition of the memory into the basolateral amygdala (BLA) which is necessary for fear memory, whereas HPC engram cells gradually became silent with time (Kitamura et al., 2017). This is an important finding, as it begins to explain how the long term storage of contextual memory remains stable, despite the observed shift in the active cell population makeup of HPC-stored representations over time (Mankin et al., 2012; Ziv et al., 2013); The hippocampal representations may need to be supported by representations constructed from engrams elsewhere in the brain, and that the maturation of the mPFC engrams over time may compensate for the decorrelation of hippocampal representations. Matos et al. (2019) demonstrated that activity of a small subset of mPFC neurons is sufficient and necessary for remote memory expression, and selective disruption of cAMP response element-binding protein (CREB) function in mPFC engram cells after CFC impairs remote memory formation (Matos et al., 2019), while another recent study suggests that the neural population in mPFC activated during memory recall changes over time (DeNardo et al., 2019). While further studies will be necessary for the understanding of mPFC engram cells for remote memory formation, these studies suggest that contextual information activates a set of neural ensembles and generates engrams in multiple brain regions during learning that are functionally connected (Kitamura et al., 2017; Roy et al., 2017b; Choi et al., 2018; DeNardo et al., 2019; Matos et al., 2019; Pignatelli et al., 2019). Recent advances in technology have the potential to generate an even deeper understanding of engram ensembles. Roy et al. (2019) applied a combination of genetic access to activated neurons labeled by the c-Fos promotor with a tamoxifen dependent Cre recombinase CreER^T2^ through the whole brain in a Fos-TRAP (targeted recombination in active populations) mouse line (Guenthner et al., 2013). All neurons activated during the CFC protocol within a narrow (< 12 h) time window were labeled with tdTomato fluorescence, then those brains were made transparent using tissue clearing techniques and imaged intact at the whole-brain scale. The mapping and subsequent optogenetic manipulations revealed new contextual engram populations in the anteromedial thalamus and the nucleus reuniens. They found that simultaneous chemogenetic reactivation of these multiple engram assemblies conferred a greater level of memory recall than reactivation of a single engram ensemble (Roy et al., 2019). Engram cells encoding CFC memory have also been identified in RSC (Cowansage et al., 2014), and optogenetic activation of those engram cells facilitates the systems consolidation of CFC memory (de Sousa et al., 2019). These results show that CFC engrams exist in brain regions other than the classically highlighted areas. Understanding the mechanisms of contextual memory at the systems level, such as ensemble activity or synchrony between brain regions will be of great interest for future studies.

## Conclusion

In this review, we have discussed the behavioral neuroscience of contextual fear conditioning in the EC-HPC network. Contextual memory has been found to be a unique part of episodic memory, encoding information about the conditions within a space in such a way as to serve as a hierarchical basis for encoding and indexing memories. Contextual information is fed to the HPC largely by Reelin^+^ cells in MEC layer II via the trisynaptic pathway. This information is then used to form unique ensembles of activity that have the ability to nest within representations of locations. Questions still remain as to the nature of the local processing of contextual data within the HPC, but recent advances in technologies for cell type specific genetic manipulations and for *in vivo* recording of cellular activity can provide powerful opportunities for investigation of these local circuit mechanisms. For example, the *in vivo* visualization of calcium transients during behavior, or the stable recording of cellular arrays across multiple brain regions simultaneously that have recently become possible have the potential to yield critical new insights into contextual learning processes as context dependent memory is composed of multiple elements, and simultaneous measurement of physiological neuronal activity could tell us which sub-component is driving the changes in neuronal activities. At the same time, utilizing cell type specific techniques to visually identify neuronal activity in specific subsets of hippocampal neurons could be of great use in further developing our understanding of the local processes that shape contextual encoding.

## Author Contributions

WM, TK, and SO conceived this project. WM, JY, TK, and SO wrote the manuscript and approved the final manuscript. WM and SO designed and created the figures. All authors contributed to the article and approved the submitted version.

## Conflict of Interest

The authors declare that the research was conducted in the absence of any commercial or financial relationships that could be construed as a potential conflict of interest.

## Publisher’s Note

All claims expressed in this article are solely those of the authors and do not necessarily represent those of their affiliated organizations, or those of the publisher, the editors and the reviewers. Any product that may be evaluated in this article, or claim that may be made by its manufacturer, is not guaranteed or endorsed by the publisher.
